# Atonic Dyspepsia1Read before the Central New York Medical Association, Buffalo, N. Y., October 16 1894.

**Published:** 1895-01

**Authors:** A. L. Benedict

**Affiliations:** Lecturer on Digestive Diseases, Dental Department, University of Buffalo; Consultant in Digestive Diseases, Riverside Hospital, Buffalo; 174 Franklin Street


### ATONIC DYSPEPSIA.[1]

By A. L. BENEDICT, A. M., M. D.,
Lecturer on Digestive Diseases, Dental Department, University of Buffalo; Consultant in Digestive Diseases, Riverside Hospital, Buffalo.

This term, a few years ago in common use, has, in the last two or three years, fallen into reproach as indicating slip-shod methods of diagnosis. At times, when circumstances have prevented thorough investigation and symptoms have pointed plainly to a dyspepsia and to a general loss of tone, the term has almost thrust itself upon me and I have used it apologetically, yet reflection fails to show why an apology was necessary.

So shrewd a clinician as Dr. Wm. Pepper has recognized atonic dyspepsia as a type and has characterized its symptoms as follows: A large, flabby, tooth-marked tongue, not necessarily coated; languid appetite; after eating, a sense of weight referred to the epigastrium; following this, distention of the epigastrium and abdomen from the development of flatus by fermentation of the gastric contents lying too long in the stomach; depending on the flatus, eructations and borborygmus; a sense of heaviness and dulness following meals; a full and, often, moderately tender abdomen; sluggish bowels; urine containing an excess of urates; lack of nourishment and, consequently, general enfeeblement and pallor; relaxation and low tone of the whole system, of which the indented tongue is an index.

The protest against the term, atonic dyspepsia, is due to a tendency, excellent in itself, to employ analytical and objective methods of diagnosis. But much may be learned from subjective symptoms, and analysis, to be of practical value, must be followed by synthesis.

It is convenient and abstractly correct to classify functional gastric disturbances into motor, secretory, sensory and absorptive, and to recognize under each head an elevation or depression of function. But we must not imagine for a moment that we have to deal with eight corresponding types of dyspepsia nor indulge the vain hope of finding remedies whose actions are as delicately shaded as the subtleties of our classification.

The sensibility of the stomach is normally so vague that it can scarcely be below par and, excluding pure neuralgia and hysterical hyperesthesia, an increase of sensibility points to some motor or

1. Read before the Central New York Medical Association, Buffalo, N. Y., October 16, 1894.

secretory trouble. Anatomists have discovered no difference between the innervation of the muscular and the glandular elements of the stomach, and we can scarcely imagine a perversion of one function without a similar change in the other. Absorption depends, first, upon perfect digestion ; secondly, upon the circulation of blood and lymph ; thirdly, upon various mysterious osmotic conditions. It is very questionable if we have any reliable test for absorption further than noting the condition of the stomach contents and the general nutrition. It is certain that we have no means of stimulating absorption without also stimulating secretion and motion. It is altogether likely that absorptive failure does not occur if digestion is perfect.

Dr. Pepper, the master of clinical medicine, although he defines atonic dyspepsia as that form in which the muscular coat is *the* element affected, immediately contradicts his definition in the typical picture which he draws. The gaseous distention is not due to a paralyzed muscular wall, but to the work of microörganisms of fermentation growing in the absence of hydrochloric acid. The sense of dulness, the loaded urine, the lack of nourishment, and consequent general enfeeblement, are due, not to muscular weakness, but to perversions of secretion and absorption.

Ewald insists on the use of the term gastriatony in the limited muscular sense, but his brief discussion, from which case reports are conspicuously absent, only confirms the belief that the condition is purely an abstraction or of phenomenal rarity.

If the secretions are perfect, what serious effect can follow from any moderate degree of gastric sluggishness ? The time at which the stomach empties itself into the intestine differs widely with individuals and with variations of diet. So long as no chemical indigestion occurs there is no disease. Neither can the lack of churning amount to much if secretion is normal, for thousands of persons in perfect health do not masticate properly, and if the gastric juice can penetrate to the center of an unchewed lump of meat, it can certainly permeate a soft mass with very little aid of muscular contraction.

I believe we may substitute for the complicated classification of gastric neuroses, which some authorities have suggested, the very simple one of atonic and irritative types, in which muscular and glandular functions fall or rise together, while the sensibility is merely symptomatic. Occasionally a unique case may arise to justify the more complicated nomenclature.

In atonic dyspepsia, there is a diminished secretion of hydrochloric acid, a delayed and insufficient peptonization of proteids, a corresponding delay in absorption and more or less sluggishness of the gastric muscle, with constipation, indicating a similar intestinal weakness.

In the opposite, irritative type, there is an increase in the secretion of hydrochloric acid, an excessive capability for proteid digestion, a delay in the conversion of carbohydrates into maltose, because ptyalin digestion is inhibited in highly acid media, more or less complaint of pain and an irritable muscular wall. This neurosis may culminate in self-digestion of the stomach ; in other words, in gastric ulcer.

In the former type, the decrease of hydrochloric acid allows the development of microörganisms, which may produce an excess of lactic, butyric and acetic acids, with gases of fermentation. Such cases, to which the term fermentative dyspepsia is applicable, though essentially atonic and sub-acid, may give rise to many sensory and motor symptoms of irritation. In some of these cases there is a predomination of one kind of ferment. Thus, I have found butyric acid persistently present, along with its cause, the bacillus butyricus. Some patients, from the relative excess of acetic acid, speak of themselves as vinegar factories. In still other cases lactic acid is in excess, and even when there is no free acid. I have often found a marked ferric chlorid test for lactates. I question whether the lactic acid, found as the result of fermentation, is chemically identical with the normal lactic acid of the preliminary stage of digestion. In fact, some recent investigations of Penzoldt have shown the reaction to be so frequently present in healthy persons after various foods and drinks, that it is very questionable whether it is not produced in the absence of lactates and whether it has any clinical significance.

Except in high grades of acute gastritis, in the late stages of chronic catarrh and cancer and, as death approaches, in almost any disease, there is no failure of rennet and pepsin, the unorganized ferments of the stomach. On the other hand, as these ferments act qualitatively rather than quantitatively, we cannot imagine them to be in excess. Thus there is no excuse for giving pepsin, except in those cases in which it is far better to administer predigested food.

The ultimate cause of atonic dyspepsia is constitutional depression. It may be due to overwork and especially to prolonged worry. Sometimes the dyspepsia is the first manifestation of tubercular poisoning. Again, there seems to be an inherent failure of the innervation of the digestive organs. Once established, the dyspepsia is, in turn, the cause of loss of strength, of mental inertia and of visceral weakness. Some degree of simple anemia is almost inevitable. The exciting cause may be an illness of any kind, the excessive use of tea, coffee or other beverages, the lack of proper food, some error in habits of eating; often it is not discoverable.

The synopsis of symptoms by Dr. Pepper needs no addition, but the results of physical examination are important. On percussion, or succussion, or by filling the stomach through the tube, dilatation will be found to be absent, though there may be some relaxation and sagging even as low as the umbilicus. Serious disease of the liver, heart and kidneys would point to the probability of gastric catarrh, which would be verified by finding an excess of mucus in the stomach contents. Chemical examination of the contents is important; in fact, a positive diagnosis of the exact form of dyspepsia can be made in no other way. If only one or two such examinations are to be made, and more are usually unnecessary, it is best to siphon out the stomach contents an hour and a half after the test meal. The preliminary stage of digestion, in which organic acids and carbo-hydrates are normally present, should be completed in an hour, but it is well to allow half an hour's leeway for the purpose of excluding slight deviations from the normal. A mixed meal of proteids, cooked starch and fat is given, comprised in a broiled steak or chop, dry bread and butter with a glassful of water. Raw starches are excluded, since they are not digested till the intestine is reached. In a normal stomach, at the end of even an hour, this test meal will be well on the way to perfect digestion. The butter and meat fat will be recognized as oil globules without rancidity, the starch will have been converted by ptyalin into maltose and absorbed, so that neither starch nor sugar will be present in the filtered stomach contents. This filtrate will also contain some albumin, a good deal of peptone and a moderate amount of propeptone, which is a transition form in the digestion of egg and plant albumin, and here represents the nitrogenous part of wheat. The various organic acids of fermentation will be absent, but there will be a strong hydrochloric acidity.

In the ordinary cases of atonic dyspepsia, there will be a moderate degree of acidity, but with hydrochloric acid reduced in quantity and, in a surprisingly large proportion of cases, the usual tests will not reveal it at all. Butyric, acetic and lactic acids, with their microörganisms, will be present. Sometimes there will be no free acid, but an abundance of lactates. When there is not a high fermentative acidity, there is nothing to prevent the digestion of starches by ptyalin, but the absorptive power of the stomach may be at fault, so that sugar will remain after the starch has disappeared. If there is much fermentation, undigested starch will also be present. The albuminoids, instead of showing well-advanced peptonization, will be principally represented by undigested albumin and moderate amounts of propeptone and peptone. But it seems that the pepsin and hydrochloric acid, having once attacked a particle of albumin, digest it pretty thoroughly before they attack other particles, so that the filtrate may not show a conspicuous lack of digestive power, whereas a simple inspection of the stomach contents demonstrates that most of the meat is untouched. Again, an abundance of peptone may mean, not that proteid digestion is good, but that absorption is weak.

The treatment consists in supplying the deficient hydrochloric acid, which is normally present in the proportion of 2:1000 ; how much is formed altogether we do not know. If we estimate the stomach contents at 500 c. c., (about a pint,) the normal amount of hydrochloric acid is 1 gram, corresponding to a dessertspoonful of the officinal dilute acid. Practically, a tenth or twentieth of this dose usually suffices, if given at the time when it is most needed, an hour after eating. If a larger quantity is necessary, it should be given in divided doses, well diluted, sipped at intervals of a few minutes, beginning three-quarters of an hour after eating. The administration of pepsin is a confession of ignorance. The patient should be urged to take common salt in abundance, since it is from chlorids that hydrochloric acid is formed.

To stimulate motor, secretory and absorptive activity, the simple bitters may be used, but I prefer strychnine in two milligram doses, given before meals in tablet and swallowed quickly to avoid the taste. Hot water is also an excellent stimulant, and it may be used to wash down the strychnine or, if the latter is not used, a weak, hot soda solution may be given to excite the opposing acid secretion and to dissolve the mucus in those cases in which an essentially functional indigestion has caused a slight catarrh. Strychnine and hydrochloric acid are usually sufficiently antiseptic to relieve any moderate degree of fermentation. If necessary, resorcin, menthol, salol, etc., may be used. I should like to urge again the use of an oily solution of menthol sprayed through the stomach tube. This treatment, which I first reported in 1892, I have found especially adapted to gastric catarrh and atonic dyspepsia with fermentation. If there is intestinal atony as well as gastric, cascara sagrada may be added to the hydrochloric acid. I do not believe in restricting the diet to any extent; it is our business not to reduce the food to the capabilities of the stomach, but to raise the digestive power to the demands of ordinary diet. Tea, coffee and coarse vegetables should, however, be interdicted. Iron and arsenic are usually indicated as general tonics, but often forbidden by the condition of the stomach. Reduced iron, the carbonate or other mild preparations should be preferred. Ordinary hygienic vigilance must be observed.

174 Franklin Street.

DISCUSSION.

Dr. A. A. Jones: I do not think that we very often find cases of sub-acidity, cases of hypochlorhydria, developing gastric ulcer, as it is so well known in the majority of cases of gastric ulcer there is hypochlorhydria. Erosions of the stomach are not uncommon in this condition designated by Dr. Benedict. But it has been found by those who have studied diseases of the stomach, and by myself in two or three cases that I have studied, that with the erosions there is almost never gastric ulcer. I mean the true, typical peptic ulcer.

Dr. A. L. Benedict: I will say, Mr. President, that Dr. Jones simply misunderstood me. I spoke of the super-acid type in contradistinction to the atonic type and the sub-acid type as culminating in gastric ulcer. I recognize that true peptic ulcer cannot grow in dyspepsia, unless it be, possibly, the exception which proves the rule.

I think our ideas with regard to lactic acid will have to be modified somewhat by a series of experiments by Penzold, which have been recently reported in a German magazine. He has found the test for lactic acid in the wash water from a perfectly healthy stomach. He has found the same test, one which is ordinarily used, after all sorts of food—plain fish, meat diet—in fact, almost everything and in a series of healthy persons. He has found that alkali and sugar, and in respect to this I can verify his tests, will give the test in the presence of lactic acid. He has also found some of the most marked tests for lactic acid. When the attempt is made to convert the lactic acid into the lactate of zinc failed utterly. So that the test does not always show the presence of lactic acid. And, furthermore, in his series of 100 cases of persons in all kinds of diet, where we have usually considered lactic acid to be present only in the preliminary stage of digestion, he finds the tests to be more marked at the end of the first hour and to continue almost up to the time of the emptying of the stomach.

Dr. Zimmer : It is always surprising to me that when gentlemen read papers on dyspepsia and indigestion and disease of the stomach, they pay so much attention to remedies and not to diet. I believe it depends more on diet than all the remedies we can possibly put into a person's stomach to overcome acid fermentation or the other products of fermentation, decomposition, and so on. There is nothing I like to get hold of better than a case of stomach trouble that some one else or that other physicians have had and seemingly have been unable to do much for. I do not depend on medicines. I give them a little medicine, it is true, because they would not have any confidence in me if I did not. But I just put that patient on a very strict and limited diet. I make him adhere to it, and tell him, if he don't want to do that, he need not come to see me. My usual diet is this : I avoid everything, as I tell them, that is of a skinny nature ; that is to say, anything that has a skin to it I want them to avoid. I will not allow them to eat a cherry ; I will not allow them to eat an apple with a skin on it; I will not allow them to eat grapes with a skin, or raisins, and I consider that cabbage is similar to that, also onions, and I usually put those patients on fresh meats, fresh fish and stale bread, eggs, milk, etc. But, as I said before, I do not depend much on medicine. I do not wash out the stomach, because I do not think it is necessary.

Dr. Jones : Mr. President, as regards Dr. Benedict's remarks, I quite agree with him that owing to recent investigations by Penzold and Boas, whose latter investigations I have incorporated in my paper, but did not read them on account of lack of time, it has been found that lactic acid is not present during any part of digestion in a healthy stomach. After many investigations, Boas comes to that conclusion, and in only one condition of the stomach is lactic acid present. That, he says, is cancer. When lactic acid, therefore, is present, Boaz considers that diagnostic of cancer. The test of lactic acid has, in my hands, been rather satisfactory, although there are certain other drugs, acids and coloring matters that will give the same reaction. But, in many of these cases, in active fermentation, not only do we mean lactic acid fermentation, but fermentation of all kinds in the stomach, that which will produce sour, very acid, irritating contents. Acetic, butyric and lactic acid fermentation go together. As regards the remarks of Dr. Zimmer, I do not think that, while we may value diet very highly indeed, that it is strictly scientific to base a diet upon anything but a study of the stomach contents.

